# Microwave Irradiation Synthesis and Characterization of Reduced-(Graphene Oxide-(Polystyrene-Polymethyl Methacrylate))/Silver Nanoparticle Nanocomposites and Their Anti-Microbial Activity

**DOI:** 10.3390/polym12051155

**Published:** 2020-05-18

**Authors:** Mohammad A. Aldosari, Khaled Bin Bandar Alsaud, Ali Othman, Mohammed Al-Hindawi, Nadimul Haque Faisal, Rehan Ahmed, Feven Mattwes Michael, Mohan Raj Krishnan, Edreese Asharaeh

**Affiliations:** 1 King Abdul-Aziz City for Science and Technology (KACST), Riyadh 12354, Saudi Arabia; aldosari@kacst.edu.sa; 2 Chemistry Department, College of Science and General Studies, Alfaisal University, Riyadh 11553, Saudi Arabia; khaled.saud@kaust.edu.sa (K.B.B.A.); aothman@alfaisal.edu (A.O.); mfalhindawi@alfaisal.edu (M.A.-H.); fmicheal@alfaisal.edu (F.M.M.); mkrishnan@alfaisal.edu (M.R.K.); 3 School of Engineering, Robert Gordon University, Aberdeen AB10 7, UK; N.H.Faisal@rgu.ac.uk; 4 School of Engineering & Physical Sciences, Heriot-Watt University, Edinburgh EH14 4AS, UK; r.ahmed@hw.ac.uk

**Keywords:** reduced graphene oxide, silver nanoparticles, microwave irradiation, PS-PMMA, anti-microbial

## Abstract

Herein, we report a facile process for the preparation of styrene and methyl-methacrylate copolymer nanocomposites containing reduced graphene oxide and silver nanoparticles ((R-(GO-(PS-PMMA))/AgNPs)) by using (i) microwave irradiation (MWI) to obtain R-(GO-(PS-PMMA))/AgNPs and (ii) the in situ bulk polymerization technique to produce RGO/AgNPs-(PS-PMMA). Various characterization techniques, including FT-IR, XPS, Raman spectroscopy, XRD, SEM, HR-TEM, DSC, and TGA analysis, were used to characterize the prepared nanocomposites. The Berkovich nanoindentation method was employed to determine the hardness and elastic modulus of the nanocomposites. The results showed that the MWI-produced nanocomposites were found to have enhanced morphological, structural, and thermal properties compared with those of the nanocomposites prepared by the in situ method. In addition, the antibacterial activity of the prepared nanocomposites against the *E. coli HB 101 K-12* was investigated, whereby an inhibition zone of 3 mm (RGO/AgNPs-(PS-PMMA) and 27 mm (R-(GO-(PS-PMMA))/AgNPs) was achieved. This indicates that the MWI-prepared nanocomposite has stronger antibacterial activity than the in situ-prepared nanocomposite.

## 1. Introduction

In recent years, the preparation of novel systems by incorporating metal nanoparticles into the polymer matrix has been used to obtain smart materials with unique physical and mechanical properties. The embedded metal nanoparticles in polymer matrixes have recently attracted great attention since they offer a high specific strength and other mechanical properties and applications in many fields, such as optics [1,2], data storage and electronics [3], biomedical applications [4,5,6,7,8,9,10,11], DNA screening [12], biosensors [13], and antimicrobial applications [14]. Enhancement of their properties depends on the type of polymer, the size distribution, and the extent of the dispersion of nanoparticles and the interfacial interaction between the filler and polymer [15]. In previous work, a facile method has been reported for the in situ preparation of PS-RGO, PMMA-RGO, and PS-PMMA copolymers containing graphene sheets [16,17,18]. Additionally, the preparation of PS-RGO/AgNP and PMMA-RGO/AgNP nanocomposites has been reported [19,20,21]. Nevertheless, there are no reports on copolymer nanocomposites that are functionalized by the in situ microwave irradiation (MWI) method with reduced graphene oxide containing silver nanoparticles (RGO/AgNPs) as filler.

In this work, the combined effect of small amounts of two filler nanomaterials (graphene and silver nanoparticles) has been studied. Graphene is known to have extraordinary structural, mechanical, thermal, optical, and electrical properties which make it an excellent two-dimensional filler nanomaterial for polymer nanocomposites for applications in various technological fields [22,23,24,25]. However, a uniform dispersion of the fillers within the polymer matrix is crucial for attaining the desired improvement in the physical and chemical properties of the polymer matrix, mainly for graphene. This is because graphene has a strong tendency to agglomerate due to intrinsic van der Waals forces. Recent studies have shown that metal nanoparticles can be supported and anchored on graphene sheets through the reduction of GO in the presence of metal salt [26]. The structural, mechanical, thermal, optical, and electrical properties and the high surface area of graphene nanosheets make it an ideal host for incorporating metal nanoparticles and thus, excellent two-dimensional filler material for polymer nanocomposites that may find applications in numerous technological fields, e.g., catalytic fuel cells, batteries, supercapacitors, etc. [27,28,29,30]. Among various polymers, polymethylmethacrylate (PMMA) is a highly transparent polymer with good mechanical characteristics and is used for various optical and medical applications [31,32]. On the other hand, polystyrene (PS) also has excellent properties, such as nontoxicity, biocompatibility, and chemical inertness [33]. The combination of these polymers (i.e., PS-PMMA) has therefore found various applications, ranging from the medical applications (i.e., bone cementing, dentistry, etc.) to automotive industry applications [34,35]. Therefore, many techniques have been developed to synthesize nanocomposite materials, including solution mixing, melt blending, microwave irradiation (MWI), and in situ bulk polymerization [17,36,37]. Despite many reports on composites of PS with PMMA or AgNPs and GO, to the best of the authors’ knowledge, no study on the combination of these components has been carried out so far.

The present work reports the synthesis of a nanocomposite of R-(GO-(PS-PMMA))/AgNPs by two distinct methods: MWI and the bulk polymerization technique. The MWI method is a fast and easy way to synthesize RGO-PS-PMMA/AgNPs, and involves supplying energy to move the molecules faster than they can relax. This, in turn, generates a high instantaneous temperature and thus increases the yield and quality of composites [38,39,40,41]. In this work, two different synthetic methods for incorporating silver nanoparticles and reduced graphene oxide sheets (RGO/AgNPs) within the copolymer matrix of PS-PMMA using the microwave irradiation method are reported. Although MWI has been previously used to reduce metal ions to generate metal NPs [38], the present work indicates that this energy-saving method is well-suited to the synthesis of graphene-supported AgNPs inside the polymer matrix. Additionally, the results obtained showed that the impact of the MWI technique for producing silver nanoparticles/graphene nanosheets within the polymer matrix leads to an enhanced thermal stability.

The goal of this work is to understand the effect of nanocomposite preparation techniques on the dispersion of nanoparticles within the polymer matrix, the thermal behavior, and the antibacterial activity of RGO-copolymer/AgNPs nanocomposites. Therefore, the newly developed nanocomposites were characterized by FTIR, XPS, Raman spectroscopy, XRD, SEM, HRTEM, DSC, and TGA methods. The obtained nanocomposites were tested for antibacterial effects against *E. coli*.

## 2. Materials and Methods

### 2.1. Materials

The chemicals used in this study, including extra pure graphite powder (>99.5%), potassium permanganate (>99%), silver nitrate, and hydrogen peroxide (30%), were supplied from Merck, while the hydrazine hydrate (HH) (80%) was provided by Loa Chemi. The monomers (i.e., styrene (S) and methyl methacrylate (MMA)) and the initiator (i.e., benzoyl peroxide (BP)) used in this study were obtained from Acros Chemical Co. and BDH Chemicals Ltd., Saudi Arbia respectively. All of the chemicals were analytical grade and used without any further purification.

### 2.2. Preparation of RGO/AgNPs

The GO was synthesized via the Hummers and Hoffman method reported in previous work [11]. Initially, the GO (400 mg) was suspended in 25 mL of H_2_O and sonicated for 30 min, followed by the addition of silver nitrate solution (80 mg, 20 wt. %) and further mixing for 30 min. The solution was then placed inside a conventional microwave oven (Kenwood MW740) after adding 40 μL of the reducing agent hydrazine hydrate (HH), and the operating conditions were set to full power (i.e., 900 W) with an on and off pulse of 10 and 20 s, respectively, for 1.2 min [16]. Following this, centrifugation was carried out using a Centurion Scientific Ltd. centrifuge operated at 5000 rpm for 15 min and the solid was dried overnight at 80 °C. AgNPs were prepared via a similar procedure, in the absence of RGO and GO.

### 2.3. Preparation of RGO/AgNPs-(PS-PMMA)

A mixture of PS/PMMA (1:1 wt. %), RGO/AgNPs (i.e., 2 wt. %), and BP initiator (i.e., 5 wt. %) was sonicated for 1 h. The mixture was then kept at 60 °C for 20 h to promote in situ free radical polymerization. Next, the product was washed several times using excess methanol and hot water. Finally, the prepared RGO/AgNPs-(PS-PMMA) nanocomposite was dried in an oven at 80 °C overnight. For comparison, a neat PS-PMMA copolymer was also prepared via a similar procedure in the absence of RGO and GO.

### 2.4. Preparation of R-(GO-(PS-PMMA)-AgNPs by MWI

A mixture of PS/PMMA (1:1 wt. %), GO (i.e., 2 wt. %), and BP (i.e., 5 wt. %) was sonicated for 1 h, and then polymerized via the in situ bulk polymerization method, as described in the previous section, to produce GO/PS-PMMA nanocomposites. Then, 40 μL HH in the presence of 0.08 g silver nitrate was added to the GO/PS-PMMA (i.e., 0.40 g) nanocomposite and sonicated for 1 h, followed by MWI reduction.

### 2.5. Material Characterization and Testing

The chemical structure properties of the prepared nanocomposites were studied using FT-IR (Thermo Scientific Nicolet-iS10) spectra between the ranges of 4000 and 500 cm^−1^. Additionally, X-ray diffraction (XRD, Philips-Holland, PW 1729) with Cu radiation was used to investigate the properties of the nanocomposites between 2θ of 5 and 100°. The X-ray photoelectron spectrum (XPS, SPECS GmbH) measurements of the nanocomposites were taken after degassing under vacuum inside the load lock for 16 h. In addition, the Raman spectra of the prepared nanocomposite were measured using a Bruker Equinox 55 FT-IR spectrometer equipped with a FRA106/S FT-Raman module and a liquid nitrogen cooled Ge detector using a 1064 nm line of an Nd:YAG laser with an output laser power of 200 mW.

The microstructural morphology of the prepared nanocomposites was investigated using a scanning electron microscope (SEM, FEI Quanta 200, FEI, Hillsboro, OR, USA). The nanocomposites were coated with gold via a sputtering system (Polaron E6100, Bio-Rad, UK). For high-resolution transmission electron microscopy (HR-TEM) images, JEOL JSM-2100F, Japan, was used with an operating voltage of 200 kV. Ethanol was employed to disperse the nanocomposite and a drop of dispersion was added to the copper grids and dried for imaging.

The thermal stability of the prepared nanocomposites was studied using thermogravimetric analyses (TGA, NETZCH 209 F1) between the temperatures of 25 and 800 °C at a heating rate of 10 °C/min under a nitrogen atmosphere (50 mL/min). Furthermore, differential scanning calorimetry (DSC, NETZCH 204 F1) was used to determine the glass transition temperature (Tg) of the prepared nanocomposites through three steps of heating. The sample was initially heated from −25 to 100 °C at a heating rate of 10 °C/min, followed by cooling from 100 to 25 °C at a heating rate of 2 °C/min, and then heated up to 350 °C. Following this, the Tg was taken at the midpoint of the transition.

Nanoindentation measurements to determine the hardness and elastic modulus were carried out using a NanoTest^TM^ system (Micro Materials, UK) with a standard diamond Berkovich indenter. For each indentation cycle, the loading and unloading lasted for 10 s, respectively, and the dwell time at each peak load was 5 s. Five measurements were carried out on each sample at the 0.1 mN (or 100 μN) load. The force-displacement (*P-h* profile) data were used to determine the hardness (*H*) and the reduced elastic modulus (*Er*). The elastic modulus (*Ei*) and the Poisson ratio (*νi*) of the diamond indenter were 1140 GPa and 0.07, respectively, whereas the Poisson ratio (*νs*) of the sample was 0.33. For the nanoindentation test, the samples were mounted on a steel disc using cyanoacrylate adhesive. The tests of all samples were conducted at room temperature (23 °C).

The antimicrobial activity of the prepared nanocomposites was studied using disk diffusion (Kirby–Bauer method). First, a specific amount of *E. coli HB 101 K-12* bacteria (i.e., 0.1 mL) was spread on nutrient agar plates. Then, 100 mg of the prepared nanocomposites was placed on the center of the nutrient agar plate. After 24 h of incubation at 37 °C, colonies were detected, and the diameter of the inhibition zone was measured.

## 3. Results and Discussion

### 3.1. FT-IR Spectra

**Figure 1:**
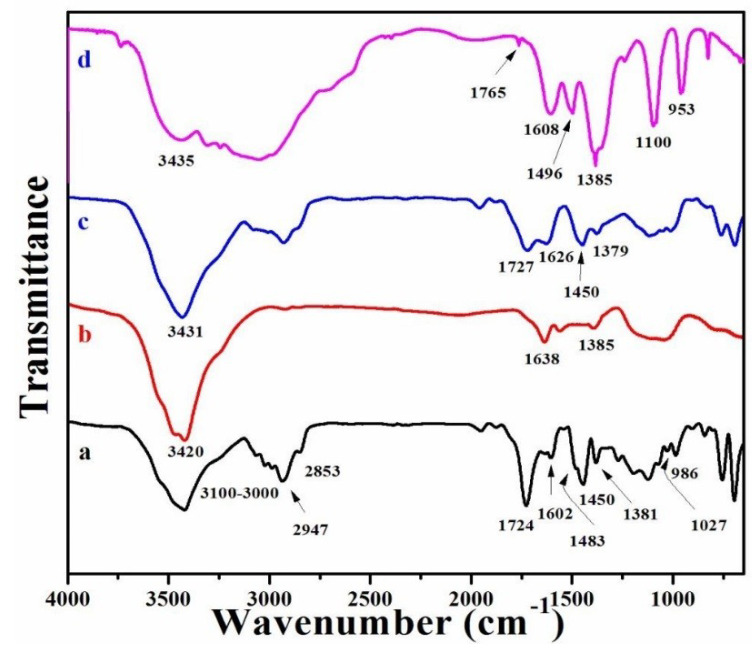
FT-IR spectra of (**a**) polystyrene (PS)-polymethylmethacrylate (PMMA), (**b**) reduced graphene oxide (RGO)/silver nanoparticles (AgNPs), (**c**) RGO/AgNPs-(PS-PMMA), and (**d**) R-(GO-(PS-PMMA)-AgNPs.

FT-IR spectral analysis was performed to confirm the chemical structure of all RGO/AgNPs copolymer composites. Figure 1 shows the FT-IR spectra of neat PS-PMMA, RGO/AgNPs, RGO/AgNPs-(PS-PMMA), and R-(GO-(PS-PMMA))/AgNPs nanocomposites. The FT-IR spectrum of the neat PS-PMMA (Figure 1a) shows the typical characteristic bands of PMMA and PS [16]. The detected peaks at 3100-3000 cm^−1^ and 1602 cm^−1^ correspond to the stretching and bending modes of –OH groups, respectively. The peaks at 2947 and 2853 cm^−1^ represent the stretching mode of C-H bonds of –CH_3_ and CH_2_ groups, respectively, while the C-H bending mode of the –CH_3_ group was detected at 1450 cm^−1^. Additionally, the peak at 1724 cm^−1^ could represent the acrylate carboxyl group (i.e., C=O) of the PMMA and the bending mode of the C=C bond for the PS. The peaks at 1381 and 986 cm^−1^ correspond to the absorption vibration of PMMA. In addition, as can be seen in Figure 1b, the FT-IR spectrum of the RGO/AgNPs nanosheet displayed the –OH groups at 3420 cm^−1^ and C=C groups at 1638 cm^−1^. Consequently, the FT-IR spectrum of RGO/AgNPs-(PS-PMMA) (Figure 1c) and R-(GO-(PS-PMMA))/AgNPs (Figure 1d) nanocomposites depicted the characteristic bands of both the neat PS-PMMA and RGO/AgNPs nanosheets. The –OH groups were detected at 3431 and 3435 cm^−1^; the C=C groups at 1727 and 1765 cm^−1^, as well as 1626 and 1608 cm^−1^; and the –CH groups at 1450 and 1496 cm^−1^, and the absorption vibration peaks of PMMA were detected at 1379, 1385, and 953 cm^−1^, for the RGO/AgNPs-(PS-PMMA) and R-(GO-(PS-PMMA))/AgNPs nanocomposites, respectively. It is important to note that the MWI-prepared polymer nanocomposite (Figure 1d) had broadened peaks with a higher intensity that slightly shifted to 3435, 1765, and 1496 cm^−1^ in comparison to the in situ-prepared polymer nanocomposite (Figure 1c). The increased intensity of the aromatic groups in the styrene (i.e., ester groups in the methyl methacrylate) could be due to π-π stacking and acrylate interaction between the RGO and polymer matrix. However, the disappearance of some peaks (i.e., 1483, 1450, and/or 1027 cm^−1^) for the polymer nanocomposites with respect to the FTIR spectrum of neat PS-PMMA could be due to the ability of the AgNPs to be stabilized by the acrylate, C=C, and O–H bonds [42]. Generally, from the FT-IR results obtained, it can be suggested that MWI promoted more interfacial interaction between the nanosheets and the polymer matrix by inducing more electron chain transfer sites.

### 3.2. XRD

**Figure 2:**
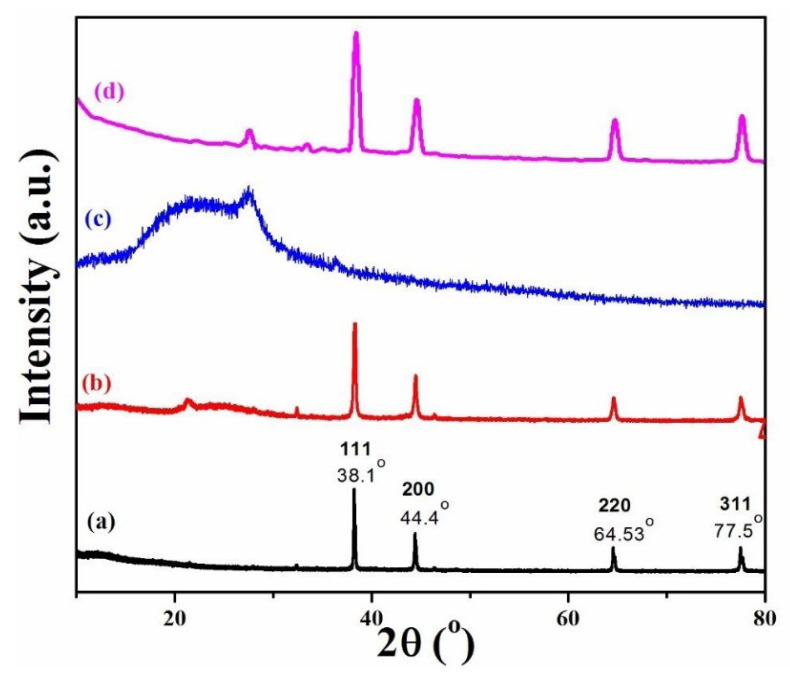
XRD patterns of (**a**) AgNPs, (**b**) RGO/AgNPs, (**c**) RGO/AgNPs-(PS-PMMA), and (**d**) R-(GO-(PS-PMMA)-AgNPs.

To carry out a further investigation of the size and structure of the RGO/AgNPs in the polymer matrix, the XRD patterns were obtained. Figure 2 depicts the XRD patterns for the prepared AgNPs, RGO/AgNPs, RGO/AgNPs-(PS-PMMA), and R-(GO-(PS-PMMA))/AgNPs nanocomposites. As can be seen from XRD (Figure 2a), the pure AgNPs show a crystalline nature with an FCC structure, exhibiting peaks corresponding to (111), (200), (220), and (311) planes. These results are in good agreement with the previous literature values for silver nanoparticles and JCPDS No.00-003-0921. The crystalline XRD pattern of the AgNPs (Figure 2a) exhibits peaks at 2*θ* values of approximately 38.1° (111), 44.4° (200), 64.5° (220), and 77.5° (311). As for the RGO/AgNPs-(PS-PMMA) nanocomposites (Figure 2c), it is difficult to find the AgNPs in the XRD pattern, as the RGO/AgNPs-(PS-PMMA) nanocomposites exhibited a broad reflection and typical amorphous nature, which could be due to the low silver concentration in the nanocomposites and the detection threshold of the XRD instrument. This could suggest that the filler (i.e., AgNPs) was inserted within the RGO/(PS-PMMA) matrix. In the case of the prepared R-(GO-(PSPMMA))/AgNPs nanocomposites (Figure 2d), the diffraction peaks of metallic Ag were detected, while the GO and RGO peaks disappeared. This could confirm the formation of AgNPs and the reduction of GO. Additionally, it could suggest that the RGO/AgNPs sheets were exfoliated within the copolymer matrix. Moreover, the lower intensity and broadening of peaks reflected the high degree of crystallinity and related to the smaller particle size of the AgNPs.

Furthermore, the average size of AgNPs and RGO/AgNPs (i.e., for the (111) peaks) was found to be ~46 and 15 nm, respectively. As for the R-(GO-(PS-PMMA))/AgNPs nanocomposites, the particle size was found to be ~7.5 nm. Meanwhile, the average size of RGO/AgNPs-(PS-PMMA) could not be calculated as the peaks were amorphous. It was also observed that the particle size was found to be smaller for R-(GO-(PS-PMMA))/AgNPs nanocomposites compared to that of AgNPs. The reduction in intensity and widening of peaks in the AgNPs/polymers (Figure 2b–d) reflect a decrease in the particle size of the polymer/AgNPs in comparison to AgNPs (Figure 2a).

### 3.3. X-ray Photoelectron Spectra (XPS)

**Figure 3:**
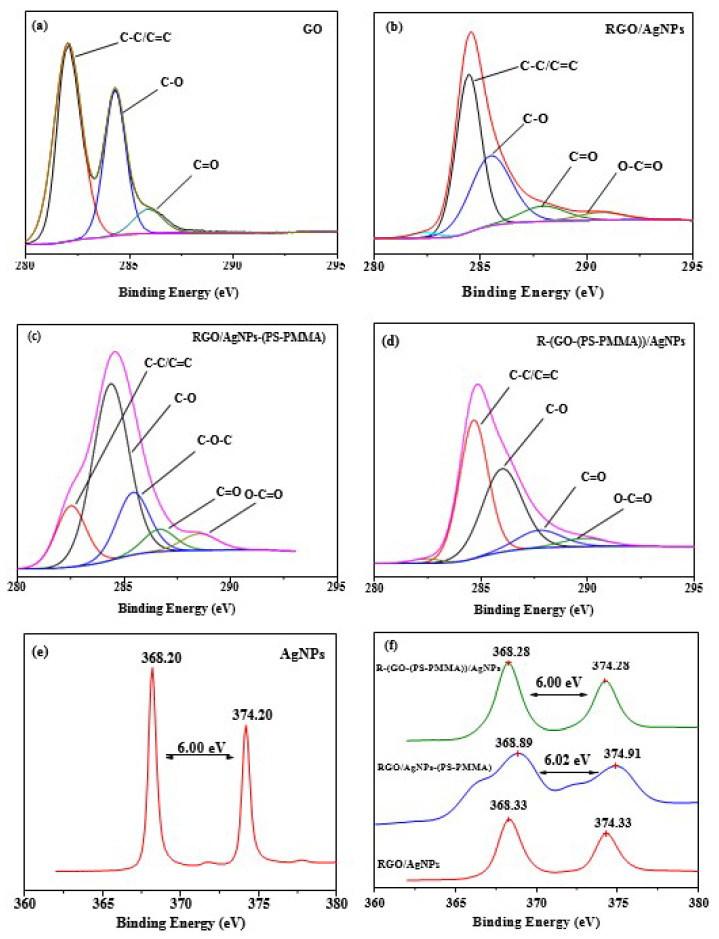
C1s and Ag3d XPS spectra of (**a**) GO, (**b**) RGO-AgNPs, (**c**) RGO-AgNPs-(PS-PMMA), (**d**) R-(GO-(PS-PMMA)-AgNPs, (**e**) AgNPs, and (**f**) the nanocomposites.

XPS was used to further study the formation of AgNPs on the surface of nanocomposites, as shown in Figure 3. This is because XPS is a powerful technique used to investigate the interaction between silver, RGO, and polymer nanocomposites. Detailed scans of Ag3d and C1s are plotted in Figure 3a–f. The C1s XPS spectra of GO (i.e., Figure 3a) displays the typical peaks at 282.0 eV for C–C, 284.3 eV for C–O, and 285.9 eV for C (epoxy/alkoxy). The peaks with a low intensity correspond to the oxygenated functionalities shown in Figure 3b–d, confirming the successful reduction of GO into RGO within the PS-PMMA matrix in the presence of hydrazine hydrate via the MWI method. Moreover, from Figure 3e,f, the peaks observed in the energy region of the Ag 3d transition are symmetric, with two characteristic binding energy peaks for Ag3d, i.e., for the metallic Ag at 374.2 and 368.2 eV corresponding to doublets of Ag3d^3/2^ and Ag3d^5/2^ [35], respectively. This indicates the metallic nature of silver and no evidence for the existence of Ag+ is obtained. Therefore, the XPS study confirmed the success of metallic AgNPs formation within the RGOPS-PMMA/Ag nanocomposites. As can be seen in Figure 3f, the XPS spectra of R-(GO-(PSPMMA))/AgNPs show the splitting of the 3d duplet at 6.00 eV. However, the in situ-prepared nanocomposite recorded splitting of the 3d doublet at 6.02 eV. The slight negative shift of about −0.02 eV suggests that there was a stronger interaction between the materials due to the MWI-enhanced electron transfer from the electron-rich RGO/PS-PMMA composites to AgNPs [42].

### 3.4. Raman Spectroscopy

**Figure 4:**
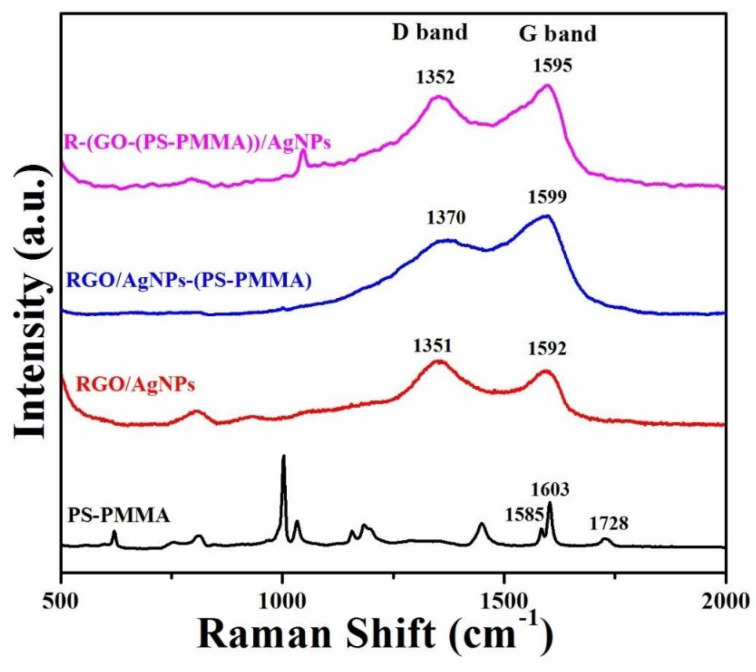
Raman spectra of PS-PMMA, RGO/AgNPs, RGO/AgNPs-(PS-PMMA), and R-(GO-(PS-PMMA)-AgNPs.

The covalent modification of RGO nanosheets can be evaluated using Raman spectroscopy [43,44]. As shown in Figure 4, the main scattered peaks for PS were observed at 1603 and 1585 cm^-1^, while for PMMA, the peak was at 1728 cm^−1^. These peaks correspond to the stretching of phenyl of the PS and the carbonyl groups (C=O) of the PMMA, respectively. Besides, the observed characteristic peaks for graphene were detected at ~1350 cm^−1^ (D band) and ~1595 cm^−1^ (G band). These D and G bands are related to the sp^3^ and sp^2^ states of the carbon group in graphene and can confirm the successful thermal exfoliation of the graphene sheets [38]. As observed in Figure 4, the intensity ratios of D/G bands are 1.46 for RGO/AgNPs, 1.09 for RGO/AgNPs-(PS-PMMA), and 1.33 for R-(GO-(PS-PMMA))/AgNPs. The attainment of a higher D/G ratio suggests better exfoliation of the graphene sheets. Therefore, the MWI-prepared nanocomposite displayed better dispersion of the RGO/AgNPs within the PS-PMMA matrix compared to that of the in situ-prepared nanocomposite. This enhancement of the D/G intensity ratio can be correlated to the conversion of sp^2^-hybridized carbon to the sp^3^ state due to the covalent interaction between RGO/AgNPs and the polymer matrix [45].

### 3.5. Scanning Electron Microscopy

**Figure 5:**
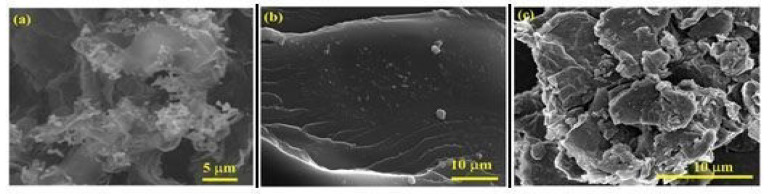
SEM images of (**a**) RGO/AgNPs, (**b**) RGO/AgNPs-(PS-PMMA), and (**c**) R-(GO-(PS-PMMA)-AgNPs.

The morphology of the RGO/AgNPs/(PS-PMMA) nanocomposites was studied using SEM, as displayed in Figure 5. The SEM image of RGO/AgNPs (Figure 5a) shows that the AgNPs were observed at the surface and embedded within RGO sheets, with good dispersion. The SEM image of RGO/AgNPs-(PS-PMMA) nanocomposites (Figure 5b) shows that the RGO/AgNPs nanosheets were dispersed within the PS-PMMA matrix. Meanwhile, for the R-(GO (PSPMMA))/AgNPs nanocomposites (Figure 5c), the AgNPs (white spots) were anchored and well-dispersed within the PS-PMMA matrix. This is in agreement with many studies that have reported incorporating RGO/AgNPs into polymer resins can improve the mechanical and rheological properties [46,47,48]. This is a result of enhanced interfacial interactions between organic polymer resins and inorganic NPs.

### 3.6. High-Resolution Transmission Electron Microscopy (HR-TEM)

**Figure 6:**
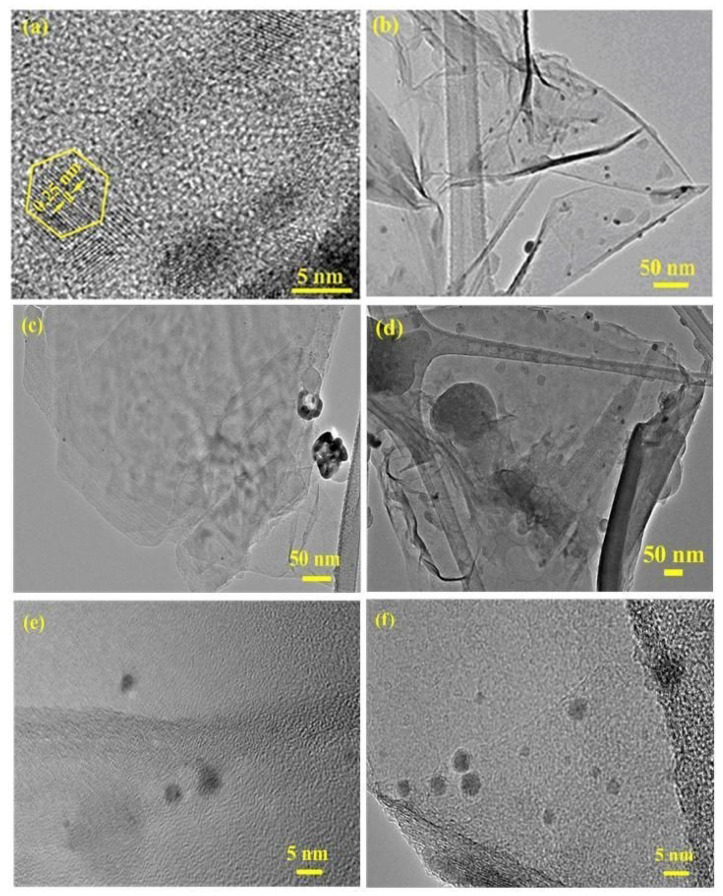
HR-TEM images of (**a**) AgNPs, (**b**) RGO/AgNPs, (**c**,**e**) RGO/AgNPs-(PS-PMMA), and (**d**,**f**) R-(GO-(PS-PMMA)-AgNPs.

HR-TEM was further used to study the surface morphology of the prepared nanocomposites and the formation of AgNPs, as displayed in Figure 6. It can be seen that the AgNPs (Figure 6a) exhibited good dispersion and small agglomeration. Additionally, the measured lattice spacing of the AgNPs was 0.25 nm, with a hexagon-shape. This indicates that these AgNPs maintained the growth of the (fcc) cubic symmetry crystal plane [34]. The HRTEM image of the RGO/AgNPs nanosheet (Figure 6b) demonstrated that the AgNPs were well-dispersed within the interface of the exfoliated RGO sheets, indicating a strong interaction between RGO and AgNPs; however, the RGO/AgNPs nanosheets were poorly dispersed within the PS-PMMA matrix (Figure 6c,e). The MWI-prepared R-(GO-(PS-PMMA))/AgNPs (Figure 6d,f) clearly showed that RGO/AgNPs nanosheets were well-dispersed within the PS-PMMA matrix. Therefore, the results of XRD, SEM, and HRTEM can verify the formation and complete dispersion of AgNPs within the RGO/PS-PMMA matrix. The uniform dispersion of AgNPs within the exfoliated RGO and PS-PMMA matrix was enhanced and improved by MWI, which should increase the thermal properties of the nanocomposites. The higher dispersion and lower aggregation resulted from van der Waals attraction among the particles, evidencing stabilization of RGO-AgNPs, which can be correlated with the electron transfer interaction between the polymers and RGO-AgNPs, in agreement with the XPS results and our previous work [19]. The particle size obtained from TEM results is in good agreement with the XRD results, which also showed a nanometer dimension.

### 3.7. Thermal Properties of the Nanocomposites

**Figure 7:**
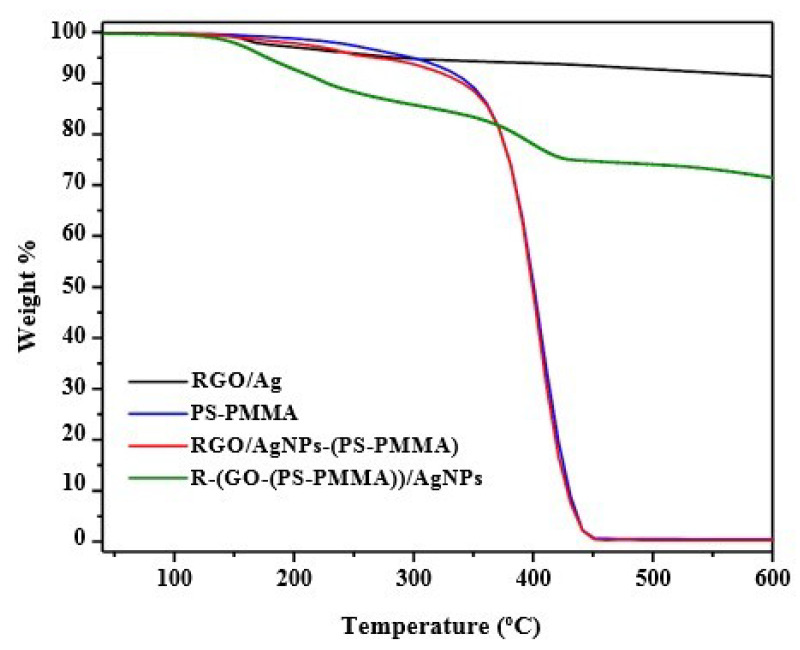
TGA thermograms for RGO/AgNPs/PS-PMMA nanocomposites.

**Figure 8:**
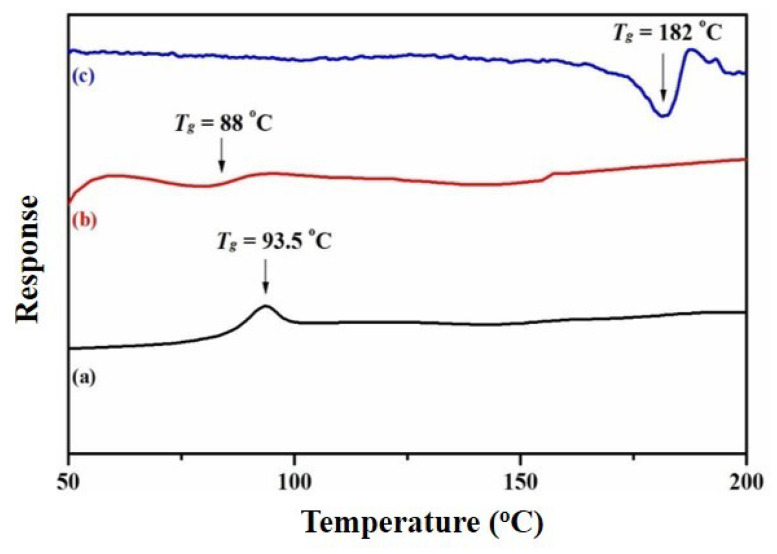
DSC thermograms for (**a**) neat PS-PMMA, (**b**) RGO/AgNPs-(PS-PMMA), and (**c**) R-(GO-(PS-PMMA))-AgNPs.

The thermal properties of the prepared nanocomposites were investigated using TGA (Figure 7) and DSC (Figure 8). The data from these analyses (i.e., TGA and DSC) are summarized in Table 1. From Figure 7 and Table 1, it can be noted that the degradation temperature of RGO/AgNPs started at over 158 °C, while for neat PS-PMMA, it began at 367 °C. However, when incorporating the nanosheets into the polymer matrix, the degradation temperatures of the polymer nanocomposites were enhanced to 400 °C for both RGO/AgNPs-(PS-PMMA) and R-(GO (PSPMMA))/AgNPs. This indicates the strong enhancement in thermal stability compared to neat PS-PMMA. In addition, the R-(GO-(PS-PMMA))/AgNPs nanocomposite clearly showed more thermal improvement than RGO/AgNPs-(PS-PMMA), as it can be noted that about 70 wt. % of the R-(GO-(PS-PMMA))/AgNPs nanocomposite was remaining at 600 °C, while the RGO/AgNPs-(PS-PMMA) nanocomposite completely degraded. The two-step thermal degradation of the nanocomposite can be correlated with the presence of AgNPs, which promoted thermal degradation of the nanocomposite in the early stages (196 °C) and caused thermal stability at higher temperatures (400 °C) [49]. This is in agreement with the SEM and TEM results reported above. As can be seen in the DSC thermogram (Figure 8), the glass transition temperatures (Tg) of RGO/AgNPs-(PS-PMMA) nanocomposites decreased by 5.5 °C with respect to neat PS-PMMA (i.e., from 93.5 to 88 °C). The Tg of the R-(GO (PSPMMA))/AgNPs nanocomposite, on the other hand, significantly improved, and a Tg of 182 °C was recorded, which is 88.5 °C higher than that of the neat PS-PMMA. This suggests and further complements the strong interaction between PS-PMMA chains and RGO/AgNPs reported through MWI. Therefore, the use of MWI is advantageous for the development of new classes of RGO/MNPs polymer-based nanocomposites.

### 3.8. Mechanical Properties of the Nanocomposites

**Figure 9:**
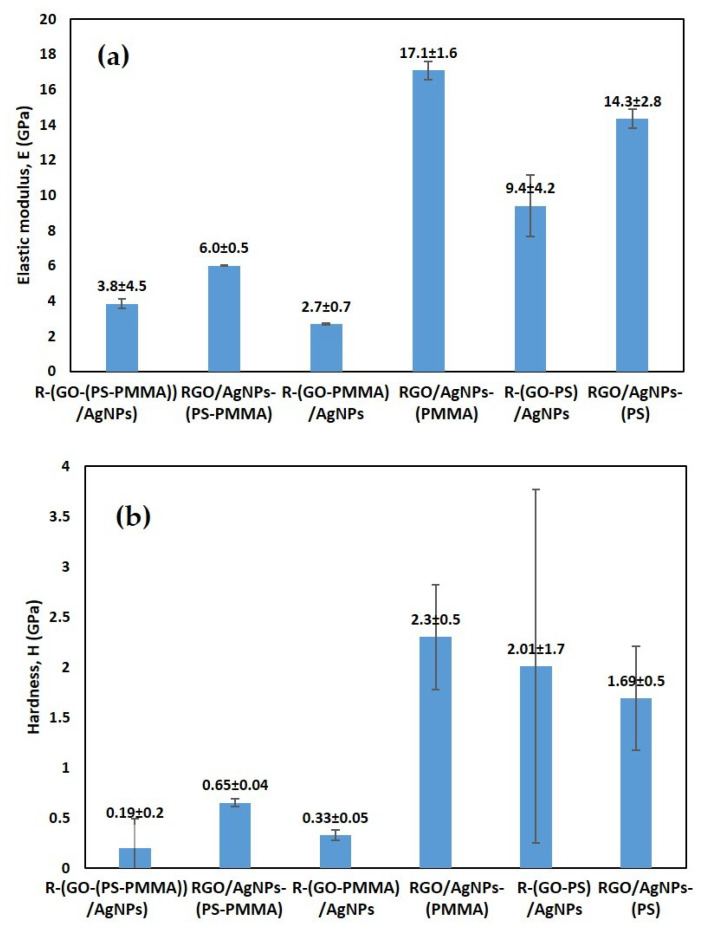
Berkovich nanoindentation: (**a**) elastic modulus and (**b**) hardness.

The hardness (*H*) and elastic modulus (*Es*) of the nanocomposites were determined from the *P–h* profiles that are shown in Figure 9 at 100 μN loads. It can be observed that the reduced graphene oxide (RGO) nano-fillers, the AgNPs-PMMA matrix, and the AgNPs-PSTY significantly improved the mechanical properties. The nanocomposite R-(GO-PMMA)/AgNPs and R (GO-PSTY)/SectionAgNPs obtained using the MWI method, however, exhibited significant degradation in the mechanical properties. The higher dispersibility and interfacial interactions between the GO nano-filler and the AgNPs-PMMA matrix were correlated with the mechanical improvements, as indicated by the dense microstructural features. These enhancements in the mechanical characteristics could be attributed to the stiffness of the nanocomposite, which arises from the number of entanglements and strong bonds within the network. Extra physical entanglements that are introduced through the cross-linking increases the overall stiffness. In the case of the R-(GO-PMMA) nanocomposite, the hardness and elastic modulus values are very similar to those of PMMA, which can be attributed to the similar Tg value and the effect of incoherent microstructural features. Based on the microstructural discussion above, the uniform distribution of AgNPs in the MWI method was expected to provide mechanical support during nanoindentation testing. However, in the case of R-(GO-PMMA)/AgNPs, the hardness and elastic modulus values were significantly low, which can be attributed to the lamella microstructure.

### 3.9. Antibacterial Activities of the Nanocomposites

**Figure 10:**
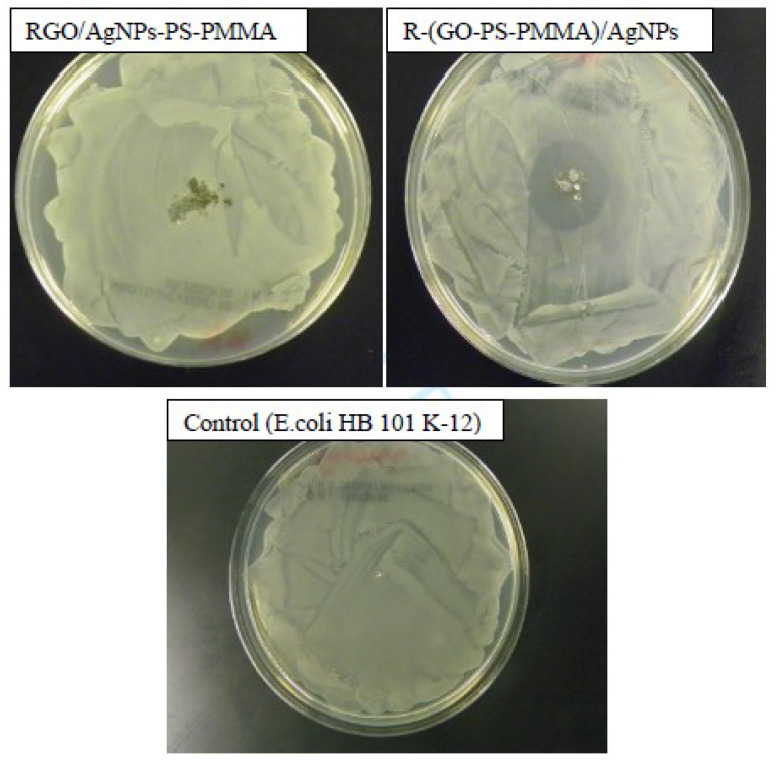
Kirby–Bauer antibacterial strength measurements for RGO/AgNPs-PS-PMMA and R-(GO-PS-PMMA)/AgNPs against *E. coli HB 101 K-12.*

The antibacterial activities of R-(GO-(PS-PMMA))/AgNPs and RGO/AgNPs-(PS-PMMA) nanocomposites against *E. coli HB 101 K-12* compared by using the diameter inhibition zone (DIZ) in the disk diffusion test are depicted in Figure 10. The nanocomposites were used as a film for antibacterial studies. It was observed that the RGO/AgNPs-(PS-PMMA) nanocomposites exhibited weaker inhibition with a 3 mm inhibition zone. The R-(GO-(PSPMMA))/AgNPs nanocomposites, on the other hand, depicted a stronger inhibition zone, with a value of 27 mm. Usually, a microorganism that is sensitive to tested samples shows a larger DIZ, compared with smaller or no DIZ for a resistant organism [6,7,8,9,10,11]. Hence, the results show that the MWI-prepared nanocomposites had stronger antimicrobial activity against the tested *E. coli* microbial strains compared to that of the in situ-prepared nanocomposite. The superior antibacterial activity of the nanocomposites prepared by the MWI method in comparison to bulk polymerization could be due to the higher mass fraction and smaller particle size of the AgNPs. This could be due to damaging proteins and genetic material that can minimize or stop bacterial growth.

## 4. Conclusions

In conclusion, in this study, RGO/AgNPs nanosheets were successfully incorporated within co-polymer matrices using in situ bulk polymerization and the microwave irradiation method. The study confirmed the success of metallic RGO/AgNPs formation within the polymer matrix. A better crystallinity of the films with smaller grain sizes was obtained using the MWI method compared with the in situ method. R-(GO-(PSPMMA))/AgNPs nanocomposites prepared by MWI are more efficient at producing covalent interactions than the in situ method. It can clearly be seen that R-(GO-(PSPMMA))/AgNPs have very small sizes of AgNPs that were dispersed at the surface and embedded within the polymer matrix. The thermal results showed a substantial enhancement in the thermal stability of the MWI-prepared nanocomposite. This can be credited to the improved dispersion of the nanosheets within the polymer matrix, which in turn enhanced the thermal stability and mechanical properties compared with the in situ-prepared nanocomposite. The antibacterial results also indicate that the nanocomposites prepared by MWI displayed strong activity against the tested microbial stains compared to those obtained without MWI. Therefore, the demonstrated properties of this newly developed nanocomposite make the material a promising candidate for the increasing demand for biomedical-related applications.

## Figures and Tables

**Table 1:** Summary of the thermal properties based on TGA and DSC measurements.

Sample	T_deg_ (°C)	T_d_ (10% Weight Loss) (°C)	T_max_ (50% Weight Loss) (°C)	T_g_ (°C)
RGO/Ag NPS	158	-	-	-
PS-PMMA	288, 367	344	397	93.5
RGO/Ag NPs-(PS-PMMA)	400	340	397	88
R-(GO-PS-PMMA)-Ag NPs	196, 400	228	<800	181
